# Clinical significance of *BRAF *mutations in metastatic melanoma

**DOI:** 10.1186/1479-5876-2-46

**Published:** 2004-12-21

**Authors:** David Z Chang, Katherine S Panageas, Iman Osman, David Polsky, Klaus Busam, Paul B Chapman

**Affiliations:** 1 Department of Medicine, Memorial Sloan-Kettering Cancer Center, New York, New York, USA; 2 Department of Epidemiology and Biostatistics, Memorial Sloan-Kettering Cancer Center, New York, New York, USA; 3 Ronald O. Perelman Department of Dermatology, New York University School of Medicine, New York, New York, USA; 4 Department of Pathology, Memorial Sloan-Kettering Cancer Center, New York, New York, USA

**Keywords:** Metastatic melanoma, *BRAF *mutation, Clinical significance

## Abstract

Forty to eighty percent of melanoma tumors have activating mutations in *BRAF *although the clinical importance of these mutations is not clear. We previously reported an analysis of *BRAF *mutations in metastatic melanoma samples from 68 patients. In this study, we correlated patient baseline characteristics, prognostic factors, and/or clinical outcomes with the presence of *BRAF *mutations. No significant differences were observed in age, gender, location of primary melanoma, stage at the diagnosis, and depth of primary tumor between patients with and without *BRAF *mutations. Melanomas harboring *BRAF *mutations were more likely to metastasize to liver (P = 0.02) and to metastasize to multiple organs (P = 0.048). Neither time to progression to stage IV nor overall survival were associated with *BRAF *mutations. In conclusion, we observed no significant differences in clinical characteristics or outcomes between melanomas with or without *BRAF *mutations. Although there was an increased frequency of liver metastasis and tendency to metastasize to multiple organs in tumors with *BRAF *mutations, there was no detectable effect on survival. Future prospective studies should include analysis of whether *BRAF *mutations in melanoma tumors correlate with an increased tendency to metastasize to liver or to multiple organs.

## Introduction

The mitogen-activated protein kinase (MAPK) pathway mediates cellular responses to growth signals and activation of this pathway has been shown to be critical in tumor formation, particularly in melanoma [1-3]. Recently, activating *BRAF *mutations were found with high frequency in malignant melanomas, including primary tumors and cell lines [4,5]. Suppression of activating *BRAF *mutations in cultured human melanoma cells inhibited the MAPK cascade causing growth arrest and promoting apoptosis [6], further suggesting the potential critical role of activating *BRAF *mutations in malignant transformation in melanoma.

We have reported the analysis of *BRAF *mutations in a cohort of metastatic melanoma patients [7] and noted a mutation proportion of 44%. As expected from previous reports, the most frequent mutation was *BRAF*^*V599E*^, which was found in 40% of samples. Since little is known about the clinical implications of activating *BRAF *mutations in melanoma tumors, we examined whether the melanoma tumors harboring *BRAF *mutations in this cohort showed different clinical or biological features compared to the melanoma tumors without mutations.

## Materials and Methods

### Retrieval of Tumor Specimens and Patient Information

Cryopreserved metastatic melanoma samples from 68 patients were selected from the Memorial Sloan-Kettering Cancer Center Tumor Bank. Patient demographic data were collected on the 68 patients whose tumors we had previously analyzed for *BRAF *mutations [7]. Data collected included: location of primary tumor, thickness, ulceration, stage of disease (according to American Joint Committee on Cancer Staging System), sites of metastasis, site of tumor biopsy, and history of and responsiveness to chemotherapy. This retrospective analysis was performed with IRB approval which determined that this was exempt research under 45 CFR 46.101.b(4).

### BRAF Mutations Detection

*BRAF *(exons 11 and 15) was sequenced as previously reported [7]. For 65/68 patients, a single metastatic site was sequenced for *BRAF*. In three patients, two to four metastatic sites were available for sequencing. For patients with multiple specimens, we considered only the first acquisition of tissue in assigning patients to mutant or wild type categories.

### Clinical Correlation and Statistical Analysis

The patients were first seen at MSKCC between June 1993 and April 2000. Clinical follow up was available through April, 2003. Comparisons between mutated and wild type were made using either the χ^2 ^test, t-test or Cochran-Armitage test to trend. Survival distributions were estimated using the Kaplan-Meier method and compared using the log-rank test. Stage IV patients were stratified into two categories: those with stage M1a or M1b (lymph nodes, soft tissues and/or lung metastasis) and those with stage M1c (all other sites).

## Results

We studied 74 cryopreserved metastatic melanoma samples from 68 patients: 42 men and 26 women (Table 1). Thirty-five patients had stage III, 33 were stage IV at the time the biopsies were obtained. These samples were melanoma metastasis from the following sites: lung (9), liver (3), gastrointestinal mucosa (9), soft tissues (20), lymph nodes (31), fallopian tube and ovarian (1), and uterus (1). Of the 68 patients analyzed, 30 had mutations in *BRAF*, including one with mutations in both *BRAF *and *NRAS*, and 38 patients were wild type. Overall, mutations in *BRAF *exons 11 and 15 were detected in 30 of 68 (44%) patients.

**Table 1:** *BRAF *mutations and clinical characteristics

**Clinical Features**	**BRAF Status**		**P value**
		
	**Mutation N = 30 (44.1%)**	**Wild Type N = 38 (55.9%)**	
**Gender**			
Female	11	15	0.81
Male	19	23	
**Age^1^**			
Mean	63.3	57.3	0.12
Median (range)	56.5 (29–91)	65.0 (42–97)	
**Stage at Diagnosis**			
I	5	3	0.92
II	13	19	
III	7	10	
IV	4	2	
Unknown	1	4	
**Thickness (Number available)**	**(N = 18)**	**(N = 22)**	
Mean	2.98	4.83	0.29
Median (range)	1.75 (0.2, 20)	2.80 (0.4, 35)	
**Primary Site**			
Head/Neck	1	6	
Trunk	10	11	
Extremities	10	14	
Ocular	1	0	
Mucosal	1	0	
Unknown	7	7	
**Response^2^**			
CR	2	3	
PR	0	2	
NR	16	10	
			
Response Rate	11%	33%	0.12

^1 ^Age at time of biopsy used to assess *BRAF *sequence.

^2 ^Response data is based on the 33 patients who received systemic therapy.

Patients' age ranged from 29 to 97 years; there was no statistically significant difference in patients' age with regards to *BRAF *mutations (p = 0.12). Similarly, there was no difference in the distribution of primary sites and stages at diagnosis between patients with and without *BRAF *mutations. We noted that among the 7 melanomas arising from the head and neck region, only 1 harbored a *BRAF *mutation. Although there were too few of these patients for a meaningful statistical analysis, this observation is consistent with a recent report indicating that mucosal melanomas did not harbor *BRAF *mutations [8,9]. The mean thickness of primary tumor was 2.98 mm (range: 0.2, 20 mm) for patients with *BRAF *mutations, and 4.83 mm (range: 0.4, 35 mm) for patients without (p = 0.29). The effect of *BRAF *mutation on other known prognostic features of primary tumor such as the presence or absence of ulceration, regression, tumor-infiltrating lymphocytes, lymph-vascular invasion, and mitotic index could not be assessed because this information was available for only a small proportion of patients.

Patients with tumors harboring *BRAF *mutations were more likely to have metastasis to liver compared to those without the mutations (41% and 13%, respectively; p = 0.02) (Table 2). Tumors with *BRAF *mutations were also more likely to metastasize to multiple organs (p = 0.048) (Table 3). Among the 51 patients who developed stage IV disease (either at the time of the biopsy or during subsequent follow up), 19 out of the 27 patients (70.4%) with *BRAF *mutations in their melanomas were found to have more than one metastatic site compared to only 11 of the 24 patients (37.5%) with wild type *BRAF*.

**Table 2:** Correlation between *BRAF *mutations and number of metastasis among patients with stage IV melanoma

**Sites of Metastasis**	**BRAF Status**		**P value**
		
	**Mutation N = 27 (%)**	**Wild Type N = 24 (%)**	
Soft Tissue/Lymph Nodes/Lung only	8 (30%)	12 (50%)	0.16
Non-soft tissue site	19 (70%)	12 (50%)	0.14
Liver	11 (41%)	3 (13%)	0.02

**Table 3:** Association of *BRAF *mutations with the number of metastatic sites in patients with stage IV melanoma

**Number of Sites Per Patients**	**BRAF Status**		P value*
		
	**Mutation N = 27 (%)**	**Wild Type N = 24 (%)**	
5	4 (14.8%)	0	p = 0.048
4	4 (14.8%)	3 (12.5%)	
3	6 (22.2%)	5 (20.8%)	
2	5 (18.5%)	3 (12.5%)	
1	8 (29.6%)	13 (54.2%)	

* Cochran-Armitage test for trend

We examined the response to systemic therapy (chemotherapy or biochemotherapy) for the 33 patients who received such treatments. For patients with *BRAF *mutations, 18 patients received systemic therapy of who two patients achieved complete remission (response rate 11.1%). Fifteen patients with wild-type *BRAF *received systemic therapy of whom three patients achieved complete remission and two achieved partial remission (response rate 33.3%) (p = 0.12).

There was no statistically significant difference between time to progression to stage IV disease either from the time of diagnosis or from stage III in patients with or without *BRAF *mutations (data not shown). As this is a retrospective study, we cannot rule out the possibility that differences in interval assessments affected our ability to detect a difference in time to progression. On the other hand, date of death is an endpoint not affected by interval assessment times. There was no statistically significant difference between patients with *BRAF *mutations and those without *BRAF *mutations.

## Discussions

High frequency of *BRAF *mutations has been reported in malignant melanoma [4,5,7], however, there has been little clinical correlation data elucidating the biological effects of these mutations in patients. We initiated this study in an attempt to address this question.

The observation that *BRAF *mutations are common in melanocytic nevi [10] has led to the assumption that mutations in *BRAF *occur early in melanocytic transformation and play an important role in the initiation of malignant transformation. Recently, an alternative view has been suggested by Dong et al who confirmed the high frequency of *BRAF *mutations present both in nevi and later stage melanomas but found few *BRAF *mutations in early stage radial growth phase melanomas [11]. They interpret these findings to mean that *BRAF *mutations are not involved in the initiation of the majority of melanoma, but rather play a role later in progression.

Since little information was available on the biological effects of activating *BRAF *mutations in melanoma, we analyzed the clinical characteristics of 68 melanoma patients whose tumors we had previously analyzed for *BRAF *[7]. We found that patients with tumors harboring a *BRAF *mutation were more likely to have metastasis to the liver and tended to have more organs involved with melanoma than patients without mutations. This is consistent with the idea that activating *BRAF *mutations affect the pattern of metastatic spread in melanoma, although we await confirmation of these findings in a prospective study.

In our cohort of subjects, there were 33 patients who received systemic therapy (18 patients with *BRAF *mutations, 15 patients without detectable mutations). There was a trend towards lower response rates among patients with mutations, although this trend was not statistically significant and is confounded by the small number of patients, the heterogeneity of treatments these patients received, and the retrospective nature of these analyses. This is a question that deserves to be revisited in a prospective manner.

Kumar and colleagues found that melanoma patients with *BRAF *mutations showed a statistically significant diminished duration of response to treatment compared to those without the mutations [12,13]. Their retrospective analysis consisted of 38 patients with metastatic melanoma (stage III or IV) who had been treated with chemoimmunotherapy (dacarbazine, vincristine, bleomycin, lomustine, and human leukocyte interferon). This cohort of patients had a surprisingly high response rate of 55%. Although the likelihood of response did not correlate with the presence of a *BRAF *mutation, multivariate analysis revealed that among patients who had responded, patients with *BRAF *mutations had a shorter duration of response compared to patients without any *BRAF *mutations (median 3.4 versus 9.8 months). They did not analyze the effect of *BRAF *mutations on the site of metastatic spread or other biological characteristics of the tumor.

Houben et al. reported that the presence of *BRAF *mutation in a metastatic melanoma lesion was associated with a poor prognosis as measured by shortened survival [14]. In our study, we did not detect any impact on either progression free or overall survival by the presence of *BRAF *mutation. The patient characteristics were not reported by Houben and colleagues but they indicate that most patients had soft-tissue metastases (M1a or M1b). In contrast, most of our patients had M1c melanoma and this could account for the different findings.

In three patients, multiple metastatic samples were available for analysis; in 2 of these patients, there was discordance in the presence of detectable *BRAF *mutations. In one patient in whom 2 lung metastasis collected over a period of one month were analyzed, one metastasis contained a *BRAF*^*V599E *^mutation; the other metastasis was wild-type for *BRAF*. In another patient, metastasis from lung, gastrointestinal (GI) tract, lymph node, and soft tissue were collected of a period of 34 months. All tumors harbored the *BRAF*^*V599E *^mutation except for the GI metastasis which was wild-type. It is possible that this discordance represents a problem with assay sensitivity, but we cannot rule out the possibility that there is true heterogenicity among metastasis with regard to *BRAF *mutations. Although this discordance among metastasis seems to contradict the observation that *BRAF *mutations are an early event in melanocytic nevi transformation, one possibility is that in melanomas arising from non-nevus melanocytes, *BRAF *mutation is a late event occurring in individual metastasis. Consistent with this, Shinozaki et al. recently reported that the incidence of *BRAF *mutation of primary melanoma did not correlate with Breslow thickness, and there was significantly higher frequency of *BRAF *mutation in metastasis than in primary melanoma, arguing that *BRAF *mutation maybe acquired during development of metastasis [15]. Houben also reported that in 3/22 cases, the *BRAF *mutational status of the primary and metastasis did not correlate [14]. This issue merits further investigation.

In summary, this analysis represents the largest study to date correlating *BRAF *mutations and clinical outcomes in metastatic melanoma. Although we observed a statistically significant higher frequency of liver metastasis and tendency to metastasize to multiple organs in patients with *BRAF *mutations, there was no significant effect on survival or response to systemic therapy detected by this study. Although this analysis is limited by its retrospective nature and the relatively small number of patients, it appears unlikely from these observations that there will be a major qualitative difference in the biological behavior between melanomas with and without *BRAF *mutations. Larger prospective studies are required to verify these observations and to clarify other biological consequences of *BRAF *mutations in melanoma.
